# Analysis of SARS-CoV-2 RNA on surfaces in New York City

**DOI:** 10.7189/jogh.11.05022

**Published:** 2021-10-02

**Authors:** Rabia Karani, Qun Zeng, Aliaa Abdelhakim, Vlad Diaconita, Omar Moussa, Henry W Zhou, Tarun Sharma, Marium Sohail, Zachary Snow, Alexis Kassotis, Angela Y Chang, Saurabh Sudesh, Stanley Chang, Jason D Horowitz, Lisa Park, Danielle Trief, Tongalp H Tezel

**Affiliations:** 1 Department of Ophthalmology, Edward S. Harkness Eye Institute, Columbia University Irving Medical Center, New York Presbyterian Hospital, New York City, New York, USA; 2 Columbia University, Vagelos College of Physicians & Surgeons, New York City, New York, USA

## Abstract

**Background:**

This study sought to determine the presence of SARS-CoV-2 virus on surfaces that trainees and faculty of an academic eye clinic came into contact with during daily life at the time of the COVID-19 pandemic in New York City.

**Methods:**

This cross-sectional analysis involved collection of at least two samples by teams on four different days (November 9, 2020 – December 18, 2020) using sterile swabs (Puritan HydraFlock, Garden Grove, CA). Collection sites were grouped into four zones depending on proximity and amount of time personnel spent there. Samples were transported to the laboratory in transport medium and RNA was extracted using the QIAamp DSP Viral RNA Mini Kit (Qiagen, Germantown, MD). Presence of viral RNA was investigated using the Luna Universal Probe One-step RT–qPCR kit (New England Biolabs, Ipwsich, MA).

**Results:**

834 samples were submitted. Two were positive for SARS-CoV-2 RNA. The first was a sample from a patient bathroom sink handle in the main emergency department. The second was a nasal swab sample from a staff member who had been assigned to collect samples. Prior to this positive result, this asymptomatic staff member had tested positive for COVID-19, had quarantined for two weeks, and had received a negative test.

**Conclusion:**

Though COVID-19 is currently widespread in the United States, this study shows that health care personnel working in New York City at the Columbia University Irving Medical Center have a low chance of encountering viral RNA on surfaces they are in close contact with during daily life.

In December 2019, an outbreak of pneumonia caused by a novel coronavirus called severe acute respiratory syndrome coronavirus 2 (SARS-CoV-2) was reported in Wuhan, China [1-3]. Infections spread across China and other countries over the following weeks [4,5]. In January of 2020, the World Health Organization (WHO) announced that the outbreak was a Public Health Emergency of International Concern (PHEIC) [6]. The disease caused by the novel coronavirus was named Coronavirus Disease 2019 (COVID-19) by the WHO and declared a global pandemic in February and March of 2020 [7,8]. In the United States, the first COVID-19 patient was reported on January 15, 2020, in Washington state. Coronavirus quickly spread throughout the United States, turning it into the global epicenter of the disease with the greatest number of cases and deaths. The first case of COVID-19 in New York was reported on February 29, 2020 and as of April 2020, New York was one of the worst hit states in the United States, with 195 749 cases making it the epicenter within the country [9].

Transmission of SARS-CoV-2 occurs primarily via respiratory droplets from face-to-face contact during talking, coughing, and sneezing. Epidemiologic data suggest that prolonged exposure to an asymptomatic infected person and brief exposures to persons who are symptomatic are associated with higher risk for transmission [10]. Aerosol spread may occur, but the role of spread in humans is unclear [11,12]. Another possible mode of transmission is contact surface spread. Studies have suggested that the virus is able to survive on the surfaces of various materials, such as plastic, metal, or glass, from two hours to nine days [13]. Given the ability of the virus to remain viable on dry surfaces for sufficient time, forward transmission has been suggested by a few studies [14-16]. Viral RNA particles were detected on the surfaces of cabins up to 17 days after patient disembarkation in a study of cruise ship outbreaks [17]. Aytogan et al. demonstrated the presence of COVID-19 viral particles using real-time polymerase chain reaction (RT-PCR) on surfaces in an eye examination room despite a triage system to exclude patients with coronavirus disease [18]. In addition, previous studies on the 2003 outbreak of severe acute respiratory syndrome (SARS) have identified viral particles on surfaces of two hospitals in Thailand and Taiwan [19].

WHO recommendations are to ensure that environmental surfaces are consistently and correctly disinfected in order to reduce viral load and the risk of transmission through contaminated surfaces. Environmental surfaces must be thoroughly cleaned with water and disinfectant. Using hospital disinfectants such as sodium hypochlorite can be an effective method [20]. Disinfectants with 62%-71% concentration of ethanol or bleach with a concentration of 0.1% sodium hypochlorite were proven to reduce coronavirus infectivity on surfaces within one minute of exposure [13]. Disinfection protocols were implemented on various surfaces around New York City, such as bus stops, subway stations, trains, and outdoor spaces. In addition, quarantine measures and travel restrictions have aided in the control of COVID-19 spread, where asymptomatic individuals who traveled out of state were required to quarantine for ten days at home except for essential workers. Multiple measures to limit the transmission of infection were implemented at Columbia University Irving Medical Center/Edward S. Harkness Eye Institute including proper personal protective equipment (PPE) for patients and staff, a pre-arrival COVID-19 screening, a pre-arrival check-in process and screening at a welcome station for COVID-19 symptoms, disinfection and cleaning throughout each day, and visitor limitations as per Centers for Disease Control and Prevention (CDC) recommendations. Despite all these precautions, risk of health care personnel’s exposure to SARS-Co-V-2 during their daily life varied depending on the time they spent in the workplace. Although the efficacy of precautions for preventing SARS-CoV-2 contamination through environmental surfaces at hospitals clinics have been well described in the past, a comprehensive study of the risk of the physicians, staff, and trainees at an academic center to come across viral RNA in their daily life along the work-home-leisure axis has not been studied. This cross-sectional study was designed to detect the presence of COVID-19 mRNA on various surfaces that workers of an academic medical center may encounter during their daily life at the time of the COVID-19 pandemic in New York City.

## METHODS

This cross-sectional analysis did not require approval by the Columbia University Irving Medical Center institutional review board because this is a non-interventional study involving surfaces and routine SARS-CoV-2 PCR tests of volunteer sample collectors. Patients or the public were not involved in the design, or conduct, or reporting, or dissemination plans of our research. Samples were collected by faculty and trainees of the Harkness Eye Institute at the Columbia University Irving Medical Center. Each member of the collection team was assigned to a specific location that was encountered by the team member in their daily life.

### Sample collection

Samples were collected by swabbing a wide surface area of a particular location using a sterile swab (Puritan HydraFlock, Garden Grove, CA). This swab was then placed into a MicroTest Tube with Transport medium (ThermoFisher Scientific R12587), refrigerated, and processed within 48 hours. Care was taken to prevent cross-contamination. Swabs were opened separately, the surface was sampled, and the swab was placed in a vial prior to proceeding to the next surface. Members of the swabbing team were tested for COVID-19 prior to starting with the collection.

Sample collection occurred in two phases. The first phase took place over two weeks from November 9-19, 2020. The second phase took place again over two weeks from December 7-18, 2020. During the second phase of sampling, sample collectors revisited the same places they had collected from in the initial phase of the study. During each phase, each location was visited twice within the same week to obtain a sample.

**Figure 1:**
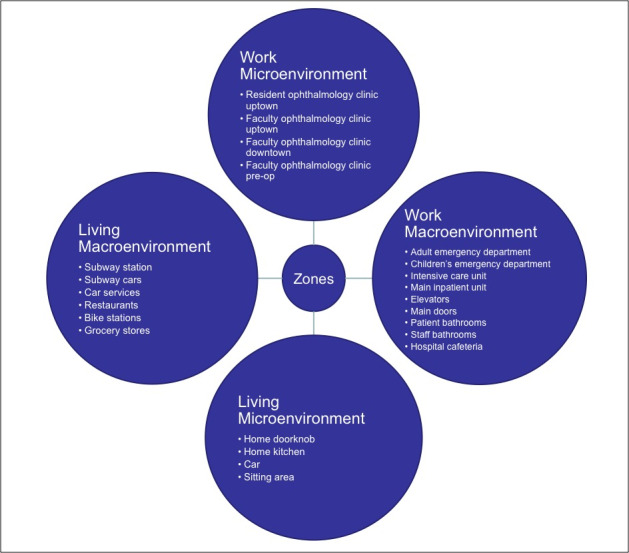
Zones for sample collection.

**Figure 2:**
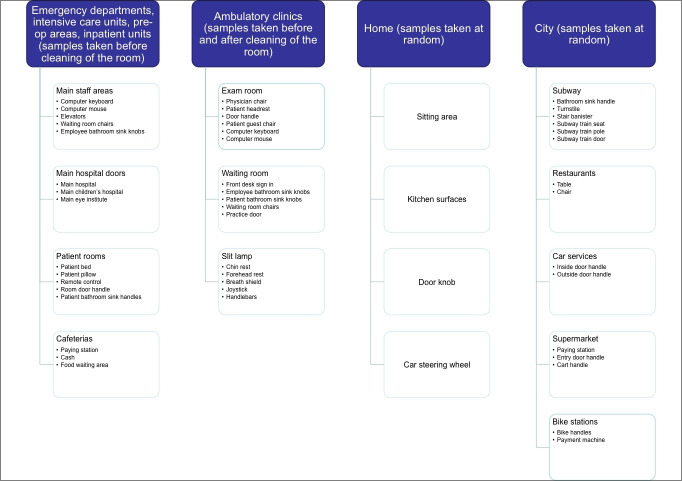
Surfaces swabbed from each zone.

Samples were collected from several different locations grouped into 4 zones (**Figure 1**). Zones included: (1) *Work microenvironment:* resident ophthalmology clinic in uptown Manhattan, faculty ophthalmology clinic in uptown Manhattan, faculty ophthalmology clinic in downtown Manhattan, ophthalmology pre-operative area. Samples in all clinics were collected before and after cleaning, and before cleaning in the pre-operative area. (2) *Work macroenvironment:* intensive care unit, children’s hospital emergency department, adult emergency department, main inpatient unit, elevators, main doors, patient bathrooms, staff bathrooms, hospital cafeteria. Samples were taken before cleaning in the inpatient units, and at random in other areas of the hospital. (3) *Living microenvironment:* home doorknob, home kitchen, car, sitting area. Samples were taken at random. (4) *Living macroenvironment:* subway station, subway cars, car services, restaurants, Citi Bike stations, grocery stores. Samples were taken at random. Surfaces swabbed in each zone included the following (**Figure 2**).

Zone 1 or clinic rooms in the work microenvironment were thoroughly disinfected with anti-viricidal wipes after every patient visit. Zone 2 or rooms in the work macroenvironment were also cleaned after each patient visit. Shared spaces such as elevators, doors, and bathrooms were cleaned daily. Zone 3 or living macroenvironment spaces were cleaned per the resident’s discretion. Zone 4 or living macroenvironment spaces were cleaned daily as well. The Metropolitan Transport Authority in New York City implemented a daily disinfection routine for subway cars and stations using antimicrobial technology and UV light technology [21]. Per CDC and New York State Department of Agriculture and Markets recommendations, many restaurants and grocery stores were cleaned daily with antimicrobials [22,23]. Citi Bike stations were also cleaned daily with antimicrobials, and car services were cleaned per the driver’s discretion [24].

### Processing

Sample processing occurred by extracting nucleic acid using a commercial viral RNA extraction kit (QIAamp DSP Viral RNA Mini Kit, Qiagen, Germantown, MD). The Luna Universal Probe One-step RT–qPCR kit (New England Biolabs, Ipwsich, MA) was then used with standardized primer and probe concentrations of 500 nM of forward and reverse primer, and 250 nM of probe, for the 2019-nCoV_N1, 2019-nCoV_N2, and RP (human control) primer–probe sets to detect SARS-CoV-2 in each sample. PCR cycler conditions were reverse transcription for 10 minutes at 55°C, initial denaturation for 1 minute at 95°C, followed by 45 cycles of 10 seconds at 95°C and 30 seconds at 55°C on CFX Connect Real Time System (Biorad).

Analysis of data was primarily descriptive.

## RESULTS

**Figure 3:**
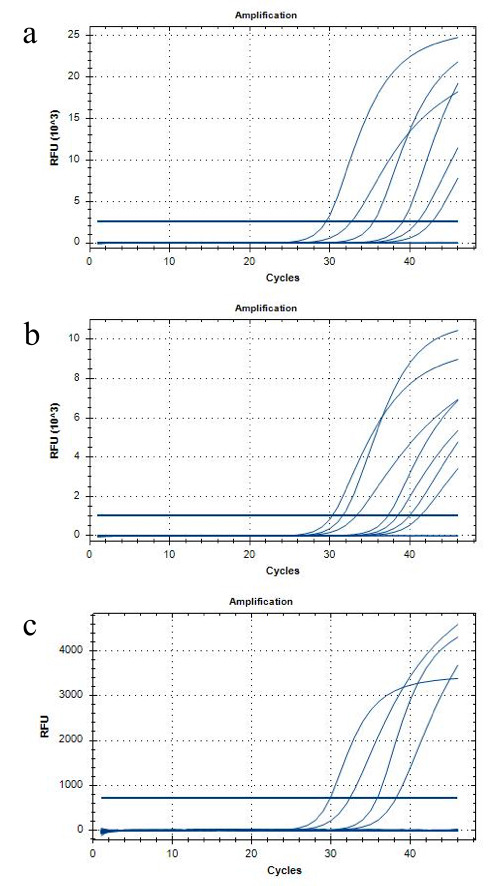
RT-PCR. RT-PCR cycles showing positivity for SARS-CoV2-mRNA from a bathroom sink handle (**Panel A**) and from a nasal swab of a participant (**Panel B**). RT-PCR cycles showing no positive results (**Panel C**).

Out of a total of 834 swabs, only two (0.24%) samples were positive. The first was a sample from a patient bathroom sink handle in the main emergency department, and the second was a nasal swab from a staff member who had been assigned to collect samples. The positive sample from the bathroom sink handle was collected on November 17, 2020 (**Figure 3**, Panel A). On this day, the effective reproduction number (R_t_) of the virus was 1.14 and the number of positive tests that day were 5088 in New York state [25]. In the emergency department of our sister hospital, New York Presbyterian Hospital Cornell, there were 864 visits related to COVID-19 in the 7-day period beginning with this day. Therefore, there were about 123 COVID-19 related ED visits on November 20, 2020 in the New York Presbyterian ED. COVID-19 related visits included patients who presented for suspected or confirmed COVID-19, or presented for testing [26]. The positive sample from the staff member was collected on December 14, 2020 (**Figure 3**, Panel B). On this day, the effective reproduction number (R_t_) of the virus was 1.10 and the number of positive tests that day were 9044 in New York state [25]. In the New York Presbyterian Hospital Emergency Department, there were 598 visits related to COVID-19 in the 7-day period centered around this day. Therefore, there were an estimated 85 COVID-19 related ED visits on December 14, 2020 in the New York Presbyterian ED [26]. Prior to this positive test result, this asymptomatic staff member had tested positive for COVID-19, quarantined for two weeks, and had received one negative test result.

The rest of the samples were negative for 2019-nCoV_N1 and 2019-nCoV_N2 primers (**Figure 3**, Panel C), although many were positive for the RP (human control) primer (**Table 1**).

**Table 1:** Areas positive for human control primer

Areas positive for human control primer	
All nasal swabs	Hospital computer mouse
Slit lamp chin rest	Hospital computer keyboard
Slit lamp forehead rest	Hospital employee bathroom sink knob
Slit lamp breath shield	Hospital patient bathroom sink handle
Slit lamp handlebars	Hospital patient room door handle
Slit lamp joystick	Hospital main elevator lobby button
Slit lamp applanation tip	Hospital floor waiting room chair
Clinic exam room patient headrest	Home swab
Clinic exam room door handle	Bike station bike handles
Clinic exam room physician chair	Bike station payment machine
Clinic exam room guest chair	Subway stair banister
Clinic room computer keyboard	Subway bathroom sink handle
Clinic employee bathroom sink knobs	Subway train seat
Clinic patient bathroom sink knobs	Subway train pole
Clinic front desk sign in	Subway door
Clinic practice door	Subway turnstile
Hospital patient bed	Subway elevator
Hospital patient pillow	Car service door handle
Hospital patient room remote control	Car service seat
Hospital patient room door handle	Main hospital door
Hospital cafeteria paying station	Supermarket cart handle
Hospital cafeteria cash	Supermarket paying station
Hospital cafeteria food waiting area	

## DISCUSSION

Though COVID-19 is currently widespread around New York City, we found evidence that there was minimal risk of exposure to COVID-19 through contaminated surfaces encountered in the daily lives of faculty and trainees at an academic medical center in New York City. Considering the 1.77%-2.39% citywide test positivity among individuals during the time this study was conducted, the likelihood of faculty and trainees being exposed to SARS-CoV-2 virus from contaminated surfaces is 100 times less likely than acquiring COVID-19 from an infected individual.

In our study, we found evidence of COVID-19 on a bathroom sink handle in the main emergency department. Several studies of COVID-19 that acquired air and surface samples taken from the rooms of COVID positive patients showed higher levels of contamination in bathrooms. Our findings were consistent with these prior studies supporting the view that viral shedding continues for a longer term from the gastrointestinal route [27,28]. Our study also found COVID-19 mRNA in the nasal passage of a study member who had already tested positive for COVID-19, had quarantined for two weeks, and had already had one negative test. All samples collected by this study team member were negative. Studies have shown that COVID-19 positivity can occur several weeks after active infection or negative testing. This can be attributed to low viral load remaining after active infection, re-infection with a new strain of virus, false positivity on RT-PCR or simply shedding of the viral RNA but not the whole virus itself. In many cases, the viral load is enough to be detected by PCR testing, but not enough for inoculation of others [29,30]. This staff member was allowed to continue swabbing.

We hypothesize that surfaces were largely negative throughout the city, in the hospital, and in the clinic environment due to strict infection control policies. All subways, restaurants, supermarkets, and other services in the city enacted strict disinfection protocols and universal masking policies. Though the virus can have surface stability for up to 72 hours, studies have shown that the use of viricidal agents was effective in eliminating human coronaviruses such as SARS and MERS, and that masking can reduce the transmission of virus by up to 90% [13,14,31,32]. In addition, the city implemented strict quarantine and social distancing policies, which were found to be important in reducing the incidence and mortality of COVID-19 [33].

In the hospital, all admitted patients were swabbed for COVID-19, and were isolated if they tested positive for the virus. Our study did not involve swabbing the rooms of COVID-19 positive patients since these areas were not the routine places the staff were visiting in their daily activities. In ambulatory care areas, patient COVID-19 status was unknown, but rooms were cleaned after each patient exam and strict screening protocols were implemented. Staff members in the hospital and in the ophthalmology clinic were known to be healthy and were screened daily, with frequent testing occurring in the case of exposure, travel, or upper respiratory or other symptoms. Visitors were also limited in the inpatient units, clinics, and emergency departments at this time, further limiting the possibility of transmission of COVID-19. One study showed that almost 4% of asymptomatic visitors to the hospital were positive for COVID-19, highlighting the importance of visitor restriction [34]. In addition, screening protocols, universal masking, strict PPE protocols, and frequent cleaning procedures were implemented throughout the different areas of the hospital and clinic per CDC guidelines [35]. A recent study at a large academic medical center demonstrated that with proper infection control protocols, the incidence of nosocomial COVID-19 in patients admitted for non-COVID reasons was 1 out of 8370 patients [36].

This study had several limitations. First, RT-PCR establishes only the presence of the viral RNA, but cannot discern the presence of the whole virus and thus, infectivity. Thus, the study cannot reach a conclusion about the risk of transmission from the positive sample that we found. Additionally, since our goal was to analyze the presence of the virus on surfaces along the work-home-leisure axis of the personnel, we excluded the rooms of COVID-19 positive patients. Obviously, physicians who consulted with these patients may have a higher risk of exposure to COVID-19. Also, the targeted viral gene and the primers that were used might have affected the ability of viral RNA detection. We used primers suggested by the CDC that were commonly used to test for the presence of SARS-CoV-2 for research purposes to minimize the impact of marker-induced sensitivity problems [37]. Finally, our samples taken from the city were gathered at random times. Although the infection rate remained stable in New York for a long time there may be variances among different times of the day which may affect the presence of SARS-CoV-2 RNA on environmental surfaces.

## CONCLUSION

Our study shows that the virus is less likely to be transmitted by contact with surfaces in New York City. Our study also shows that decontamination, universal masking, visitor restriction, PPE protocols, quarantine protocols, and outdoor dining and recreation limit the spread of the virus. Finally, our study further bolsters the hypothesis that the virus has mainly respiratory transmissibility.
